# The effect of western diet on mice brain lipid composition

**DOI:** 10.1186/s12986-019-0401-4

**Published:** 2019-11-27

**Authors:** Alicja Pakiet, Agnieszka Jakubiak, Aleksandra Czumaj, Tomasz Sledzinski, Adriana Mika

**Affiliations:** 1 Department of Environmental Analytics, Faculty of Chemistry, University of Gdansk, Wita Stwosza 63, 80-308 Gdansk, Poland; 2 Tri-City Academic Laboratory Animal Centre - Research & Services Centre, Medical University of Gdansk, Gdansk, Poland; 3 Department of Pharmaceutical Biochemistry, Medical University of Gdansk, Debinki 1, 80-211 Gdansk, Poland

**Keywords:** Eicosapentaenoic acid, Docosahexaenoic acid, brain, High-fat diet, Mice model, Sphingolipids, obesity, Polyunsaturated fatty acids, Phospholipids

## Abstract

**Background:**

The appropriate fatty acids composition of brain lipids is critical for functioning of this organ. The alterations of brain fatty acids composition may lead to neurological and neurodegenerative diseases.

**Methods:**

The aim of this work was to evaluate the effect of western diet containing high fat content on fatty acid composition of brain lipids. In this study we used mice fed high fat diet (HFD) for 19 weeks. Brain lipids were separated by SPE extraction and fatty acid composition in chow, mice serum, brain and other tissues was analyzed by GC-MS method.

**Results:**

The body weight and adipose tissue weigh of mice after HFD increased significantly. The concentrations of most of fatty acids in serum of mice after HFD increased, due to their higher delivery from food. Unexpectedly the serum eicosapentaenoic acid (EPA) concentration was lower in mice after HFD than in controls. Also the brain, and other tissue EPA content was lower. Among studied groups of brain lipids EPA was significantly decreased in phospholipids and sphingolipids.

**Conclusions:**

Considering important role of brain EPA including maintaining of appropriate composition of cell membrane lipids and anti-inflammatory properties we conclude that decrease of brain EPA after western diet may result in impaired brain function.

## Background

Western diet is characterized by reduced intake of n-3 polyunsaturated fatty acids (PUFA), high saturated FAs and n-6 PUFA intake, as well as elevated levels of refined sugar and overuse of salt [1]. The above mentioned factors contribute to the epidemic of obesity, one of the world’s major public health problems [2]. More than two in three adults in the United States are obese or overweight [3, 4]. Also, increased rates of cancer, increased inflammation, reduced control of infection, and increased risk for allergic and auto-inflammatory disease are one of many negative effects of Western diet. Fascinatingly, some of our poor dietary behaviors have a genetic background and are passed to our offspring [5].

For decades, the health-promoting properties of long chain n^− 3^ PUFA were well known. The properties of the another PUFA family, n-6, are also familiar. These all fatty acids are cellular modulators and precursors of sphingolipids, oxylipins and endocannabinoids [6]. Endocannabinoids belong to a class of lipids derived from arachidonic acid (ARA, 20:4 n-6). They participate in the control of many physiological functions in the body, including food intake, energy balance, and reward [7]. Additionally, endocannabinoids are capable of neuromodulation and are presumably involved in mood regulation and vulnerability to psychosis [8]. What is important, it was proven, that high-fat diet (HFD) leads to elevations in peripheral endocannabinoid signaling [7]. Synaptamide (N-docosahexanoyloethanolamine, DHEA), a docosahexaenoic acid (DHA) endocannabinoid-like metabolite, is capable of inducing neuronal differentiation of neural stem cells by induction of phosphorylation of protein kinase A (PKA)/cAMP response binding element (CREB) [9]. Further, alterations in the levels of n-6 PUFAs oxidation products (eicosanoids) in brain is associated with schizophrenia, epilepsy, multiple sclerosis, Alzheimer’s, Parkinson’s disease [10]. However, protectin D1, oxylipin formed from DHA, has anti-apoptotic and neuroprotective properties [8] and can stimulate neural differentiation of embryonic stem cells [11]. Another long chain n-3 PUFA, eicosapentaenoic acid (EPA) presents anti-inflammatory properties [12, 13]. There is a huge research showing that EPA, DHA and also metabolites generated from these acids has pro-healthy properties. In turn elevated n-6/n-3 ratio leads to disorders in the functioning of the organism, mainly inflammation [14], as well as psychiatric disorders, including severe depression [15], schizophrenia [16, 17] or anxiety disorders [18]. In turn, many studies showed positive impact of EPA and DHA on the treatment of depression [12, 19]. Their large amounts exist in neutral phospholipids and modulate the dopaminergic and serotonergic pathways [12]. But, the benefits of EPA and DHA are not limited to prevention of depression. Supplementation with EPA and DHA of moderately malnourished school-aged children yielded in improvement of IQ, executive functioning, visuoperceptive capacity and processing speed [20]. Another study of n-3 PUFA supplementation in healthy older adults showed their beneficial effects on cognition [21], dementia [22] and the brain [21]. Also the intake of n-3 PUFA prevented psychosis in adolescents [23]. Long chain n- 3 PUFA are essential for retinal, neuronal and immune system development of fetus [24]. Summarizing, EPA and DHA due to their neuroprotective properties can be used in a treatment in neurological and neurodegenerative diseases [25].

## Materials and methods

The aim of our work was the determination of polyunsaturated fatty acids levels in brain, and in other organs of mice fed high fat diet (HFD), which is the equivalent of a human western diet.2.1 Animals and treatment.

Twenty-six-week-old males C57BL/6 mice from Tri-City Animal Laboratory Research and Service Center, Medical University of Gdansk, were randomly assigned for two experimental groups (average body weight 21,8 g). Each mouse had individual marking. The first group was fed a normal diet containing 10% fat (Altromin, ME 14.6 MJ/kg), while mice from second group were fed high fat diet containing 60% of fat (Altromin, ME 21.1 MJ/kg). Animals were housed for 18 weeks in polysulfone cages in temperature 22 ± 2 °C, humidity 55 ± 10%, 12-h dark-light cycle, with air exchanged 12 times or more per hour. Mice were fed ad libitum and had free access to water. Body weight and feed intake were measured weekly. At the end of the experiment mice were sacrificed and blood as well as brain, liver, kidney, muscle and subcutaneous, epididymal and retroperitoneal adipose tissue samples were collected. Blood was centrifuged at 3000×g for 15 min at 4 °C, and serum was stored at − 80 °C. Tissue samples were immediately frozen in liquid nitrogen and stored at − 80 °C until analysis.

### Lipids analysis

#### Sample preparation

Extraction of total lipids from tissues and serum was carried out with a mixture of chloroform:methanol (2:1, v/v) as described by Folch et al. [26], chloroform phase was collected and dried under nitrogen stream. Next, the lipid extracts were divided into two parts: for SPE extraction and analysis of fatty acids (FA) profile from total lipid samples.

#### SPE extraction

Tissue samples were fractionated according to two procedures, which differed with regards to collected lipid fractions. Procedure I, described by Kaluzny et al. [27], yielded free fatty acids (FFA), polar lipids/phospholipids (PL) and acylglycerols (AG). Briefly, 2 mg of tissue extracts prepared with Folch et al. [26] method were dissolved in chloroform and loaded on aminopropyl cartridges (Strata® NH2 500 mg, Phenomenex®) preconditioned with 4 mL of *n*-hexane. Next, the lipids were eluted as follows: 6 mL chloroform:isopropanol (2:1, v/v) - neutral lipids (NL), 6 mL diethyl ether:acetic acid (98:2, v/v) – FFA, 6 mL methanol – PL. These fractions were saved and evaporated to dryness. NL were then reconstituted in *n*-hexane and loaded on secondary aminopropyl cartridge as described above. Subsequently, the column was eluted with 6 mL *n*-hexane - cholesteryl esters, discarded, 9 mL diethyl ether:methylene chloride:*n*-hexane (1:10:89, v/v/v) – triacylglycerols (TAG), 18 mL ethyl acetate:*n*-hexane (5:95, v/v) – cholesterol, discarded, 6 mL ethyl acetate:*n*-hexane (15:85, v/v) – diacylglycerols (DAG) and 6 mL chloroform:methanol (2:2, v/v) – monoacylglycerols (MAG). The acylglycerols (AG) fractions were than combined and dried under nitrogen stream.

Procedure II followed Bodennec et al. [28] method. 1.5 mg of tissue extracts prepared with Folch et al. method was reconstituted in chloroform and loaded on aminopropyl cartridges (Strata® NH_2_ 500 mg Phenomenex®) preconditioned with 5 mL *n*-hexane. Then, the samples were eluted using 5 mL ethyl acetate:*n*-hexane (15:85, v/v) – neutral lipids without ceramides (Cer), MAG and FFA, 4 mL chloroform:methanol (23:1 v/v) – Cer, 3 mL diisopropyl ether:acetic acid (98:5, v/v) – FFA and α-hydroxy-FFA (α-OH-FFA), 11 mL acetone:methanol (9:1.35 v/v) – glycosphingolipids (GSPL) and chloroform:methanol (2:1, v/v) – sphingomyelins (SM). The eluates were evaporated to dryness.

#### Hydrolysis step

Obtained samples from I and II SPE procedures, as well as total lipid samples, were then hydrolyzed with 1 mL of 0.5 M KOH in methanol at 90 °C for 3 h. Subsequently, the mixture was acidified with 0.2 mL 6 M HCl. Next, 1 mL of water was added, and FA were extracted three times with 1 mL of *n*-hexane and dried under nitrogen stream.

#### GC-MS analysis

FA after hydrolysis were derivatized using 10% boron trifluoride-methanol solution at 55° in order to prepare FA methyl esters (FAME). After 1.5 h 1 mL of water was added to the mixture and FAME were extracted three times with 1 mL of *n*-hexane and dried under nitrogen stream. Prepared FAME were analyzed with GC-EI-MS QP-2010SE (Shimadzu, Japan). The FAME separation was conducted on Zebron ZB-5MSi capillary column (30 m length × 0.25 mm i.d. × 0.25 μm film thickness). The GC oven temperature was set at 60–300 °C (4 °C/min) with overall run time of 60 min. Helium was used as carrier gas with the column head pressure of 100 kPa. Mass spectrometry detection was performed with an electron impact source operating at 70-eV. Mass spectra acquisition was conducted with full scan mode with mass scan range m/z 45–700. 19-methylarachidic acid was used as an internal standard. FA were identified using reference standards (37 FAME Mix, Sigma-Aldrich) and reference library NIST 2011.

### Statistical analysis

For normally distributed data the statistical significance of differences between means was estimated by using parametric (Student’s T), whereas the data without normal distribution were analyzed by nonparametric (U Mann-Whitney, Wilcoxon) tests. Data is presented as means ± SD. All calculations were performed using Sigma-Plot 11 software (Systat Software, Inc., 2008).

## Results

### Induction of obesity by HFD

**Fig. 1:**
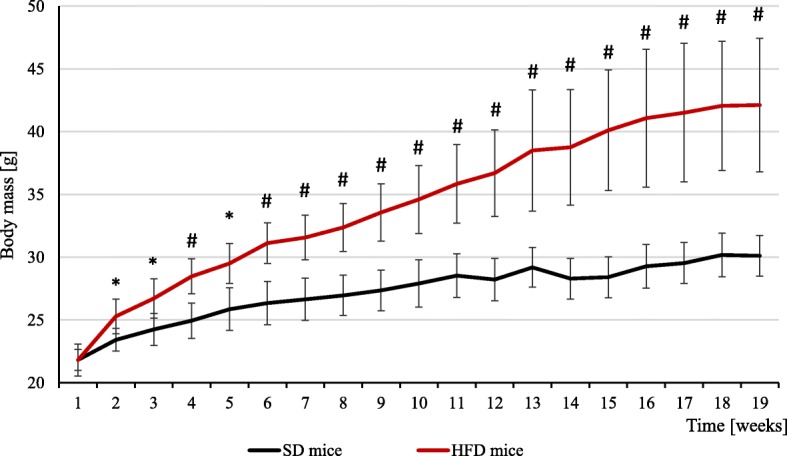
The body weight of mice with obesity induced by high fat diet and control mice. HFD – high fat diet; SD – standard diet. *p*-value: * < 0.05; # < 0.001

The treatment of mice by HFD (containing 60% fat) for 19 weeks resulted in significant increase of body weight comparing to controls fed standard diet for laboratory mice (SD) including 10% fat (Fig. 1). The statistically significant difference was seen beginning from the second month of treatment (Fig. 1). After 19 weeks of experiment, we observed about 30% increase of mean body mass of HFD mice (Table 1). There were no significant changes in the mass of brain, heart and liver, however the non-statistical tendency for higher mass of liver in HFD mice can be observed (Table 1). All adipose tissue (AT) depots were much heavier in HFD mice than in controls and also the kidney weights were significantly higher (Table 1).

**Table 1:** Masses of organs/tissues in lean and obese mice fed by HFD after 19-week treatment

	SD mice organ/tissue mass [mg]	HFD mice organ/tissue mass [mg]
Brain	372 ± 43	378 ± 38
Liver	1322 ± 203	1394 ± 447
Retroperitoneal adipose tissue	94 ± 30	762 ± 361^*^
Epididymal adipose tissue	308 ± 70	1846 ± 549^#^
Subcutaneous adipose tissue	246 ± 30	1010 ± 548^*^

*p*-value: * < 0.05; # < 0.001, SD – standard diet mice, HFD – high-fat diet mice

### Effect of HFD on FA content in serum and selected mice organs

**Fig. 2:**
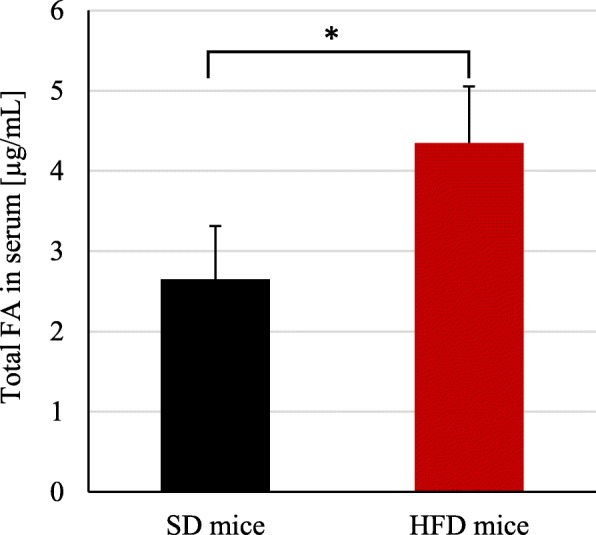
The level of total fatty acids in serum of SD and HFD mice. HFD – high fat diet; SD – standard diet. *p*-value: * < 0.05

The concentrations of all n-3 PUFA (excluding EPA) as well as n-6 PUFA were higher or similar in serum of HFD mice comparing to controls (Table 2), that is probably associated with higher content of each PUFA in high fat chow (Table 2). Surprisingly, we observed two-fold decrease of EPA concentration in serum of HFD mice, despite the fact that both control mice and mice with induced obesity consumed chow containing the same proportion of all n-3 PUFA in their diets, and the amounts of all n-3 PUFA were higher in high fat chow (Table 2). Also, lower concentrations of EPA than DHA in serum of both SD and HFD mice were detected (Table 2). The total fatty acids in HFD mice was almost twice-fold higher than in SD group (Fig. 2).

**Table 2:** Composition of polyunsaturated FAs in major tissues

	Chow [μg/kg]		Serum [μg/L]		Brain [%]		Liver [%]		Retroperitoneal adipose tissue [%]		Epididymal adipose tissue [%]		Subcutaneous adipose tissue [%]	
	10%	60%	SD	HFD	SD	HFD	SD	HFD	SD	HFD	SD	HFD	SD	HFD
**SFA**	**205.08**	**671.00**	**971.92 ± 253.18**	**1766.61 ± 353.61**^**#**^	**46.13 ± 1.06**	**46.82 ± 2.14**	**37.41 ± 0.86**	**38.03 ± 4.72**	**33.75 ± 2.46**	29.73 ± 1.64^*^	**29.08 ± 1.53**	**29.28 ± 1.63**	**31.99 ± 1.39**	28.80 ± 2.29^*^
**MUFA**	**131.09**	**455.75**	**1079.97 ± 340.71**	**1227.99 ± 244.16**	**32.35 ± 2.64**	**30.99 ± 2.75**	**41.26 ± 2.56**	**39.15 ± 11.23**	**62.51 ± 2.16**	**62.06 ± 1.73**	**66.86 ± 1.29**	62.58 ± 1.43^*^	**64.19 ± 1.44**	**63.03 ± 2.17**
16:2 n-6	–	–	0.10 ± 0.02	0.40 ± 0.15	0.01 ± 0.00	0.01 ± 0.01	0.003 ± 0.000	0.020 ± 0.010^*^	0.01 ± 0.00	0.01 ± 0.01	0.01 ± 0.00	0.01 ± 0.00^*^	0.003 ± 0.000	0.02 ± 0.01^#^
LA	25.83	95.00	329.16 ± 24.65	870.96 ± 105.11^#^	0.38 ± 0.08	0.72 ± 0.08^#^	6.40 ± 0.46	11.56 ± 2.34^*^	3.02 ± 0.28	7.46 ± 0.10^#^	3.42 ± 0.51	7.51 ± 0.31^#^	3.16 ± 0.17	7.51 ± 0.15^#^
20:2 n-6	0.89	0.40	3.39 ± 0.48	8.88 ± 2.05^#^	0.08 ± 0.02	0.15 ± 0.03^*^	0.09 ± 0.01	0.17 ± 0.05^*^	0.07 ± 0.01	0.14 ± 0.03^#^	0.05 ± 0.01	0.11 ± 0.02^#^	0.05 ± 0.01	0.11 ± 0.02^#^
DGLA	0.26	1.25	29.86 ± 5.51	34.00 ± 13.69	0.35 ± 0.04	0.38 ± 0.01	1.35 ± 0.11	0.65 ± 0.17^#^	0.08 ± 0.02	0.06 ± 0.00^*^	0.05 ± 0.01	0.03 ± 0.02^*^	0.07 ± 0.01	0.04 ± 0.01^*^
ARA	0.48	1.75	140.57 ± 30.17	313.94 ± 49.40^#^	7.93 ± 0.61	8.11 ± 0.64	8.19 ± 0.87	6.28 ± 2.83	0.10 ± 0.03	0.10 ± 0.02	0.06 ± 0.02	0.04 ± 0.03	0.11 ± 0.02	0.07 ± 0.02^*^
AdA	0.11	0.75	1.37 ± 0.20	2.81 ± 0.534^*^	2.06 ± 0.25	2.01 ± 0.25	0.14 ± 0.02	0.17 ± 0.05	0.02 ± 0.01	0.01 ± 0.00^*^	0.01 ± 0.01	0.01 ± 0.00	0.01 ± 0.01	0.01 ± 0.00
**PUFA n-6**	**27.76**	**103.13**	**509.63 ± 50.28**	**1232.70 ± 99.52**^**#**^	**11.22 ± 0.96**	**11.52 ± 0.64**	**16.62 ± 1.38**	**18.93 ± 5.24**	**3.30 ± 0.32**	**7.79 ± 0.09**^**#**^	**3.61 ± 0.53**	**7.72 ± 0.23**^**#**^	**3.42 ± 0.18**	**7.77 ± 0.19**^**#**^
ALA	0.07	0.25	5.24 ± 1.80	9.52 ± 2.24^*^	0.02 ± 0.01	0.02 ± 0.01	0.10 ± 0.03	0.18 ± 0.06^*^	0.01 ± 0.01	0.01 ± 0.01	0.01 ± 0.00	0.01 ± 0.01	0.01 ± 0.01	0.01 ± 0.00
ETA	–	–	2.53 ± 0.43	2.37 ± 0.65	–	–	0.06 ± 0.01	0.03 ± 0.01^*^	0.01 ± 0.00	0.01 ± 0.00	0.01 ± 0.00	0.01 ± 0.01	0.01 ± 0.00	0.01 ± 0.00
EPA	0.19	0.50	39.66 ± 9.47	18.94 ± 2.29^#^	0.33 ± 0.03	0.15 ± 0.02 ^#^	1.71 ± 0.12	0.31 ± 0.11^#^	0.12 ± 0.02	0.03 ± 0.00^#^	0.13 ± 0.02	0.05 ± 0.04^#^	0.12 ± 0.02	0.02 ± 0.01^#^
DPA	0.19	0.63	2.61 ± 0.39	7.59 ± 1.90^#^	0.36 ± 0.05	0.31 ± 0.06	0.23 ± 0.04	0.32 ± 0.09	0.03 ± 0.01	0.02 ± 0.01	0.02 ± 0.01	0.01 ± 0.00	0.03 ± 0.01	0.01 ± 0.00^#^
DHA	0.04	0.25	28.40 ± 6.74	68.66 ± 7.68^#^	8.91 ± 0.57	9.71 ± 0.78	2.36 ± 0.29	2.85 ± 1.28	0.03 ± 0.01	0.01 ± 0.01^*^	0.02 ± 0.00	0.01 ± 0.01	0.02 ± 0.00	0.01 ± 0.00
**PUFA n-3**	**0.48**	**1.63**	**78.43 ± 17.61**	108.08 ± 8.18^*^	**9.62 ± 0.60**	**10.19 ± 0.79**	**4.46 ± 0.37**	**3.69 ± 1.50**	**0.20 ± 0.04**	**0.08 ± 0.01**^**#**^	**0.19 ± 0.01**	0.10 ± 0.06^*^	**0.18 ± 0.02**	**0.06 ± 0.02**^**#**^

*p*-value from t-test: * < 0.05, # < 0.001, AdA – adrenic acid (22:4 n-6), ALA – α-linolenic acid (18:3 n-3), ARA – arachidonic acid (20:4 n-6), DGLA –dihomo-γ-linolenic acid (20:3 n-6), DHA – docosahexaenoic acid (22:6 n-3), DPA – docosapentaenoic acid (22:5 n-3), EPA – eicosapentaenoic acid (20:5 n-3), ETA – eicosatetraenoic acid (20:4 n-3), LA – linoleic acid (18:2 n-6), MUFA – monounsaturated fatty acids, PUFA – polyunsaturated fatty acids, SFA – saturated fatty acid

Boldface, major groups of fatty acid

The biggest statistically significant differences in EPA content were noted in liver and they were also considerable in three adipose tissues depots (Table 2). Serum saturated fatty acids (SFA) levels were higher in HFD mice, whereas the difference in monounsaturated fatty acids (MUFA) were not statistically significant (Table 2). Moreover, the content of SFA were significantly lower in retroperitoneal and subcutaneous adipose tissue, whereas MUFA were lower in epididymal adipose tissue, heart and kidney. Most of other n-3 and n-6 PUFA contents were increased or not changed in the studied tissues. Only the level of dihomo-γ-linolenic acid (DGLA) was reduced in liver muscle and all adipose tissue depots, whereas ARA content was reduced solely in subcutaneous adipose tissue (Table 2). We also observed, that in adipose tissues EPA was the most abundant FA among n-3 PUFA, which was not the case in the liver. It was probably an effect of high concentrations of EPA in used chow (Table 2). Liver is a main organ of the FA metabolism, whereas an adipose tissue mainly uptakes FA from circulation and stores them in the form of TAG.

### Alterations of EPA content in brain after HFD diet

The most abundant PUFA among total FAs in mice brain was DHA followed by ARA. EPA was present in significantly lower amounts (Table 2). The content of EPA was more than 2-fold lower in brain of mice fed HFD and, most importantly, it was the only one PUFA, which content was lower in brain of HFD than in SD mice.

**Fig. 3:**
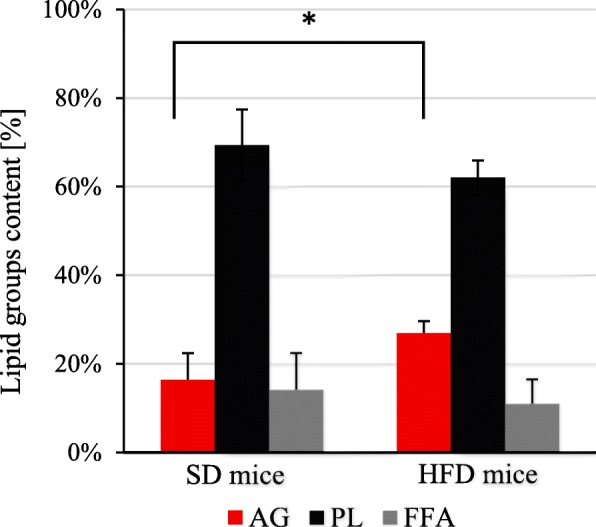
Composition of polar lipids in mice brain obtained with Kaluzny et al. [27] method. AG – acylglycerols; HFD – high fat diet; PL – polar lipids; SD – standard diet; FFA – free fatty acids. p-value from t-test: # < 0.001

Lipids are very diverse compounds group and each of them plays various functions. Therefore, we separated lipids from brain of mice on several lipid groups. One of the used chemical procedure of lipid fraction separation was Kaluzny et al. [27] SPE method that allow to obtain three lipid fractions, acylglycerols (AG), polar lipids/phospholipids (PL) and free/non-esterified fatty acids (NEFA). Percentage share of particular lipid fractions in HFD mice were changed in comparison to SD mice (Fig. 3). We found significantly higher AG fraction in brains of the HFD mice (Fig. 3). The content of PL in SD mice brain after Kaluzny et al. [27] separation, the amounts to almost 70% of lipids, and were slightly lower in HFD mice brain, but not statistically significant (Fig. 3).

**Fig. 4:**
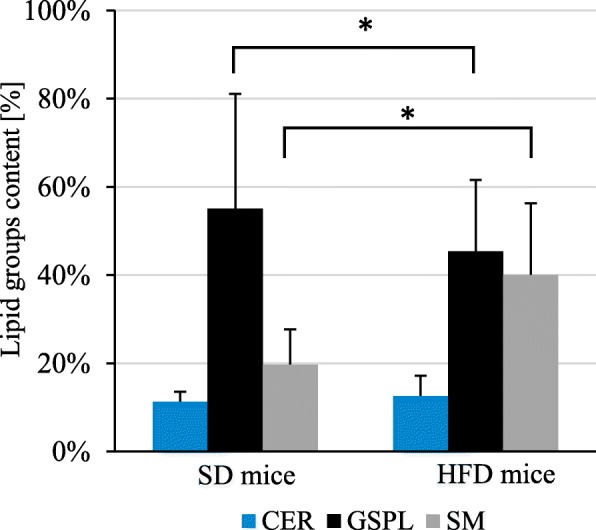
Composition of polar lipids in mice brain obtained with Bodennec et al. [28] method. CER – ceramides; GSPL – glycosphingolipids; HFD – high-fat diet; SD – standard diet; SM – sphingomyelins. p-value from t-test: * < 0.05

Second method of separation of brain lipids was modification of Bodennec et al. [28] method. By using this method, we received six fractions, including a) neutral lipids, b) ceramides (Cer), c) normal and α-hydroxy free fatty acids, d) neutral glycosphingolipids (GSPL), e) sphingomyelin (SM) and f) sphingosine 1-phosphate, ceramide 1-phosphate, and sulfatides. In our study we focused on Cer, GSPL and SM, due to their pivotal function in the brain [29]. We found significantly decreased GSPL fraction, whereas increased SM fraction in mice brains after HFD comparing to controls. The brain content of Cer was similar in both groups (Fig. 4).

Then, we analyzed the FA composition in separated fractions of lipids from mice brains (Table 3). The highest level of EPA among lipid fractions in the brains of SD mice was observed in PL fraction, and only in this fraction among those obtained by Kaluzny et al. [27] SPE method the content of EPA was statistically lower in HFD mice brain (Table 3). In acylglycerols there was no difference in EPA content between brains of SD and HFD mice, whereas in FFA fraction the content of EPA was even higher in HFD mice. Interestingly, despite that EPA and DHA belong to long chain n-3 PUFA, their participation in particular brain lipid fraction is very different. The most abundant PUFA in PL fraction was DHA, which level was significantly elevated in HFD brain (Table 3). In GSPL and Cer fraction the levels of EPA and DHA were significantly lower than ARA level (Table 3). Also, in contrast to DHA and ARA, in each determined sphingolipid (SPL) fraction we observed significantly decreased levels of EPA in brain of HFD mice (Table 3).

**Table 3:** Fatty acid content [%] in various lipid fractions in mice brain

	Procedure I (Kaluzny et al. [27])						Procedure II (Bodennec et al. [28])					
	Acylglycerols		Polar lipids		Free fatty acids		Ceramides		Glycosphingolipids		Sphingomyelin	
	SD	HFD	SD	HFD	SD	HFD	SD	HFD	SD	HFD	SD	HFD
**SFA**	**47.21 ± 5.59**	**45.13 ± 4.99**	**48.88 ± 1.00**	**48.84 ± 0.96**	**84.36 ± 1.46**	**83.68 ± 1.98**	**63.19 ± 3.16**	**57.55 ± 4.87**	**58.47 ± 1.68**	**56.96 ± 0.85**	**43.15 ± 2.55**	**44.52 ± 2.44**
**MUFA**	**33.73 ± 1.38**	36.39 ± 1.61^*^	**32.06 ± 1.89**	**29.99 ± 2.20**	**9.83 ± 1.39**	**9.79 ± 1.27**	**27.42 ± 2.06**	**30.32 ± 2.11**	**32.10 ± 1.57**	**31.85 ± 1.26**	**25.19 ± 2.43**	**23.22 ± 2.92**
16:2 n-6	0.03 ± 0.02	0.09 ± 0.08	0.00 ± 0.01	0.00 ± 0.00	0.0005 ± 0.0005	0.002 ± 0.002	0.04 ± 0.01	0.02 ± 0.02	0.01 ± 0.00	0.00 ± 0.00	0.01 ± 0.00	0.01 ± 0.00
LA	14.96 ± 5.40	14.13 ± 5.68	0.26 ± 0.02	0.46 ± 0.04^#^	2.38 ± 0.13	1.94 ± 0.55	0.53 ± 1.34	0.84 ± 0.44	0.28 ± 0.02	0.59 ± 0.12^*^	0.23 ± 0.02	0.40 ± 0.09^*^
20:2 n-6	0.10 ± 0.04	0.08 ± 0.03	0.08 ± 0.00	0.16 ± 0.03^#^	0.12 ± 0.08	0.02 ± 0.02	0.09 ± 0.02	0.09 ± 0.04	0.07 ± 0.03	0.15 ± 0.01^*^	0.07 ± 0.02	0.10 ± 0.03
DGLA	0.13 ± 0.03	0.10 ± 0.05	0.30 ± 0.03	0.34 ± 0.04	0.02 ± 0.01	0.08 ± 0.04	0.19 ± 0.05	0.25 ± 0.07	0.19 ± 0.02	0.24 ± 0.03^*^	0.34 ± 0.02	0.36 0.03
ARA	2.70 ± 0.99	2.72 ± 0.56	6.95 ± 0.86	7.44 ± 0.84	2.69 ± 0.27	3.36 ± 0.02	4.64 ± 1.08	5.54 ± 1.20	4.19 ± 0.53	4.90 ± 0.80	9.79 ± 0.79	9.66 ± 0.61
AdA	0.15 ± 0.05	0.19 ± 0.05	2.18 ± 0.32	2.21 ± 0.19	0.09 ± 0.00	0.16 ± 0.00	0.34 ± 0.07	0.73 ± 0.58	0.55 ± 0.06	0.58 ± 0.07	4.17 ± 0.41	3.86 ± 0.46
**PUFA n-6**	**18.10 ± 5.03**	**17.37 ± 5.14**	**10.12 ± 1.31**	**10.73 ± 0.96**	**5.32 ± 0.03**	**5.53 ± 0.53**	**7.09 ± 1.12**	**8.04 ± 2.08**	**5.43 ± 0.55**	**6.50 ± 0.91**	**15.17 ± 1.21**	**14.57 ± 0.85**
ALA	0.07 ± 0.06	0.04 ± 0.01	0.02 ± 0.01	0.02 ± 0.01	0.01 ± 0.00	0.02 ± 0.02	0.06 ± 0.04	0.03 ± 0.01	0.02 ± 0.02	0.02 ± 0.02	0.01 ± 0.00	0.02 ± 0.00^*^
EPA	0.18 ± 0.09	0.15 ± 0.08	0.31 ± 0.03	0.13 ± 0.01^#^	0.11 ± 0.02	0.29 ± 0.03^*^	0.34 ± 0.08	0.18 ± 0.12^*^	0.13 ± 0.01	0.06 ± 0.02^#^	0.46 ± 0.03	0.22 ± 0.05^#^
DPA	0.07 ± 0.02	0.06 ± 0.03	0.34 ± 0.05	0.35 ± 0.05	0.03 ± 0.03	0.03 ± 0.00	0.09 ± 0.06	0.11 ± 0.03	0.10 ± 0.02	0.11 ± 0.03	0.54 ± 0.05	0.48 ± 0.07
DHA	0.52 ± 0.17	0.68 ± 0.06	7.99 ± 0.61	9.77 ± 0.66^*^	0.37 ± 0.06	0.60 ± 0.11	1.58 ± 0.29	3.58 ± 1.50^*^	3.63 ± 0.42	4.41 ± 0.15^*^	15.38 ± 1.17	16.86 ± 0.92
**PUFA n-3**	**0.85 ± 0.25**	**0.93 ± 0.14**	**8.65 ± 0.66**	**10.27 ± 0.68**^**#**^	**0.51 ± 0.10**	**0.93 ± 0.09**	**2.08 ± 0.35**	3.91 ± 1.54^*^	**3.88 ± 0.44**	4.61 ± 0.14^*^	**16.39 ± 1.19**	**17.58 ± 0.97**

*p*-value from t-test: * < 0.05, # < 0.001, AdA – adrenic acid (22:4 n-6), ALA – α-linolenic acid (18:3 n-3), ARA – arachidonic acid (20:4 n-6), DGLA –dihomo-γ-linolenic acid (20:3 n-6), DHA – docosahexaenoic acid (22:6 n-3), DPA – docosapentaenoic acid (22:5 n-3), EPA – eicosapentaenoic acid (20:5 n-3), ETA – eicosatetraenoic acid (20:5 n-3), LA – linoleic acid (18:2 n-6), MUFA – monounsaturated fatty acids, PUFA – polyunsaturated fatty acids, SFA – saturated fatty acids

Boldface, major groups of fatty acid

## Discussion

### Alterations of EPA content in brain after HFD diet

The most important and at the same time surprising result of this study is significant reduction of EPA in serum, brain and other tissues in HFD compared to SD mice. Simultaneously, every other PUFA were increased or not changed in serum and brain of the HFD mice, possibly due to their higher concentration in high-fat chow (Table 2). Jansen et al. [30] suggested that mice with obesity have a higher needs for essential fatty acids than non-obese animals. EPA and DHA are the subjects of enzymatic oxidation, which results in the formation of eicosanoids and docosanoids, named oxylipins, which have anti-inflammatory properties. EPA is a precursor of three series of eicosanoids and related peroxy-fatty acids, a molecules with most powerful anti-inflammatory properties [6, 25]. In turn, ARA derived eicosanoids displays a pro-inflammatory properties [29, 31]. Thus, decreased EPA in brain may result in increase of inflammation in this organ. Another, important function of long chain PUFA is impact on membrane flexibility, fluidity and permeability as well as, assure the passive transport by the membrane [6, 25, 32]. They are main compounds of brain membranes. DHA and ARA together are almost one fifth of the brain dry weight [6, 29]. The composition of membrane lipids appears to influence the mental health, what relies on lipid-protein interactions within the membrane. It may influence neurotransmitter release and reuptake, and membrane receptors function [33]. Supplementation of EPA enhances proliferation in neural stem cells [25]. However, when EPA enters into the brain it is rapidly oxidized [34, 35], in contrast to DHA [36]. This is not the case with DHA and ARA. Infusion of ^14^C-EPA in situ showed that EPA has a half-life only 5 days in brain phospholipids, while DHA and ARA have 33 and 42 days, respectively [37]. Therefore, the research on ADHD, depression and brain trauma, suggests that EPA supplementation should be even higher than DHA to maintain its appropriate concentration in blood, and thus accessibility for the brain [38].

### Alterations of particular lipid fraction in mice brain after HFD diet

In this study for the first time the profile of fatty acids in particular lipid fractions in brain of mice treated by HFD was investigated. One of the used chemical procedure of lipid fraction separation was Kaluzny et al. [27] SPE method that allowed to obtain three lipid fractions: AG, PL and FFA. Participation of particular lipid fractions in HFD mice were changed in comparison to SD mice brain (Fig. 3). In HFD mice we found statistically significant increase of AG fraction (Fig. 3), that is mainly constituted by diacylglycerols (DAG) and triacylglycerols (TAG) [39]. The elevated levels of AG can be attributed to higher level of MUFA in HFD mice (Table 3). Also, the increased levels of acylglycerols were observed by Borg et al. [39], in the hypothalamus of HFD mice comparing to animals fed low fat diet. DAG is a product of metabolism of both phospholipids and TAG, and it is implicated in the development of central insulin resistance in the brain [39].

PL are about 70% of lipids separated by Kaluzny et al. [27] SPE method, but there was no significant difference between SD and HFD mice (Fig. 3). PL are extensively synthetized in the brain [40], constituting the majority of structural lipids located in the phospholipid bilayer [29]. The level of EPA in PL fraction in HFD brain was almost three time lower compared to SD mice brain (Table 2). By contrast we observed significant increase of EPA in HFD mice brain among FFA fraction (Table 3). Perhaps, free EPA originate from hydrolysis of PL and SPL, where its content is decreased. Interestingly, despite EPA and DHA belong to long chain n-3 PUFA, their participation in specific/particular lipid fraction is very different. DHA is most abundant in polar lipids, which are building cellular membranes of brain [25], in order to facilitate transport through the mitochondria membrane and decrease the levels of mitochondrial reactive oxygen species [41]. DHA is about 40% of all PUFAs in the brain [42, 43], what was also observed in our study (Table 2). DHA due to additional double bond in its structure, and longer aliphatic acid than EPA, takes up a lot more space in membrane, which results in the greater fluidity of brain membranes. This allows for more effective receptor functioning and signal transmission into the interior of the nerve cells [44].

Second method of separation of brain lipids used in our study was modification of Bodennec et al. [28] method. In this way we received five fractions, including neutral lipids, Cer, normal and α-hydroxy free fatty acids, GSPL and sphingomyelin. In our study we focused on SPL, due to their important functions in the brain [29]. Sphingolipids is a wide lipid group including Cer, SM and GSPL [29]. SM and GSPL are mainly localized in the plasma membrane, acting as determinants of membrane permeability and fluidity [45]. Concentration of sphingolipid in brain is very high due to their function in creation the lipid rafts [29]. GSPL in their structure have various carbohydrates attached to the ceramide [29], and one third of the GSPL are gangliosides, which in neuronal membranes constitute 12% of total lipid content [46]. Their function in membrane is protein modulation, synaptic transmission, cell-cell adhesion, neural development and differentiation, axonal growth, and receptor regulation [47]. Perhaps, that is why they were in the highest levels among examined sphingolipids in the brains of examined mice (Fig. 4). GSPL levels in HFD mice were significantly decreased that may lead to alterations of the above mentioned important function in the brain In turn, elevated levels of Cer in HFD brain compared to SD brain (Fig. 4) is associated with lipotoxicity of Cer including induction of apoptosis, inflammation and central insulin resistance in the brain [39].

Dominant PUFA among SM fraction was DHA, which plays a significant role in cells signaling as a component of lipid rafts [48]. Also, due to DHA greater flexibility it is more readily incorporated into the glycosphingolipids membrane [49]. In GSPL and Cer fraction the levels of EPA and DHA were significantly lower than ARA level (Table 3). In contrast to DHA and ARA, in each determined sphingolipid fraction we observed significant decrease of the levels of EPA in brain of HFD mice (Table 3). One reason for decreased EPA content in brain of mice after HFD may be its lower availability in blood. Moreover, the other reasons of lower EPA levels can be the involvement of EPA in lipid metabolism in the brain, including β-oxidation, elongation/desaturation to docosapentaenoic acid (22:5n-3; DPA), which is precursor of DHA [50]. EPA is such an important acid not only due to the above-mentioned properties, but also affects the functioning of the brain. Studies on rat brains showed that EPA is increased in cortical tissues, improved spatial memory in the aged rats and restores log-term potentiation [51]. The above-mentioned data suggest that decreased levels of EPA in PL, and sphingolipids in brain of HFD mice may contribute to brain dysfunction.

### Changes of PUFA/EPA in other mice organs

Consumption of food reach in fat leads to fat gain and increased body weight, especially diets containing more than 30% of total energy as fat lead to the development of obesity [52]. However, it has been reported that not every fat is obesogenic and the fatty acid profile rather than the energy from fat is crucial in the development of obesity [52]. On the other hand, some studies did not show differences between body-weight gain of the animals consuming food containing various fatty acids [52].

Some authors described elevated levels of TAG and FFA in serum mice after high-fat diet [53, 54]. TAG and FFA are responsible for induction of oxidative stress, lipotoxicity, dyslipidemia, insulin resistance and diabetes [55]. Also, our study showed significantly increased serum total fatty acids, that are possibly included both in TAG as well as FFA fractions. The excessive deposition of lipids in cell other than adipocytes leads to cellular stress, dysfunction, and sometimes to apoptotic cell death termed lipotoxicity. This process is involved in the development of many diseases [39]. The fatty acid composition of various lipids is often reflective of the fatty acid composition in consumed food [56]. However, despite that the concentration of total FA in high fat chow was four time higher than in standard chow (Table 2), the levels of EPA were significantly decreased in serum and all analyzed HFD mice organs (Tables 2, 3).

Significant differences in EPA content in liver and three adipose tissues depots (Table 2) may also lead to increase of adiponectin secretion in adipose area [57], that could increase the risk of comorbidities of obesity, including cardiovascular disease and insulin resistance [58]. What is more, adequate amount of EPA in intake diet prevents obesity by inducing mitochondrial biogenesis and beta-oxidation in adipocytes [59].

## Conclusions

Our study showed that in contrast to other PUFA, the western diet caused significant decrease of EPA content in mice serum, brain and other tissues. In the brain the decrease of EPA was significant among phospholipids and sphingolipids, that are essential components of cell membranes. Decreased EPA in the brain after HFD may be a result of lowered availability of this FA from blood or its conversion into other FA in brain cells. Decreased EPA levels in brain may lead to increased inflammation, structural changes in cell membranes and consequently to the impaired brain function.

## Data Availability

Data sharing is not applicable to this article as no datasets were generated or analyzed during the current study.

## Abbreviations

AG: Acylglycerols
ARA: Arachidonic acid
AT: Adipose tissue
Cer: Ceramides
DAG: Diacylglycerols
DGLA: Dihomo-γ-linolenic acid
DHA: Docosahexaenoic acid
DPA: Docosapentaenoic acid
EI: Electron ionization
EPA: Eicosapentaenoic acid
FA: Fatty acids
FAME: Fatty acids methyl esters
FFA: Free fatty acids
GC-MS: Gas chromatography-mass spectrometry
GSPL: Glycosphingolipids
HFD: High fat diet
MAG: Monoacylglycerols
MUFA: Monounsaturated fatty acid
NEFA: Non-esterified fatty acids
NL: Neutral lipids
PL: Polar lipids/phospholipids
PUFA: Polyunsaturated fatty acids
SD: Standard diet
SFA: Saturated fatty acids
SM: Sphingomyelins
SPE: Solid-phase extraction
TAG: Triacylglycerols
α-OH-FFA: α-hydroxy free fatty acids
