# Correction to: The effect of western diet on mice brain lipid composition

**DOI:** 10.1186/s12986-020-00444-0

**Published:** 2020-04-16

**Authors:** Alicja Pakiet, Agnieszka Jakubiak, Aleksandra Czumaj, Tomasz Sledzinski, Adriana Mika

**Affiliations:** 1 Department of Environmental Analytics, Faculty of Chemistry, University of Gdansk, Wita Stwosza 63, 80-308 Gdansk, Poland; 2 Tri-City Academic Laboratory Animal Centre - Research & Services Centre, Medical University of Gdansk, Gdansk, Poland; 3 Department of Pharmaceutical Biochemistry, Medical University of Gdansk, Debinki 1, 80-211 Gdansk, Poland

**Correction to: Nutr Metab**

**https://doi.org/10.1186/s12986-019-0401-4**

The original version of this article [1], published on 27 November 2019, contained incorrect calculations. In this Correction the affected part of the article is shown.

1. Incorrect calculations in Table 2 Composition of polyunsaturated FAs in major tissues;

**Table 2:** Composition of polyunsaturated FAs in major tissues

	Chow [μg/kg]	Serum [μg/L]	Brain [%]	Liver [%]	Retroperitoneal adipose tissue [%]	Epididymal adipose tissue [%]	Subcutaneous adipose tissue [%]							
	10%	60%	SD	HFD	SD	HFD	SD	HFD	SD	HFD	SD	HFD	SD	HFD
**SFA**	**205.08**	**671.00**	**971.92 ± 253.18**	**1766.61 ± 353.61**^**#**^	**46.13 ± 1.06**	**46.82 ± 2.14**	**37.41 ± 0.86**	**38.03 ± 4.72**	**33.75 ± 2.46**	29.73 ± 1.64^*^	**29.08 ± 1.53**	**29.28 ± 1.63**	**31.99 ± 1.39**	28.80 ± 2.29^*^
**MUFA**	**131.09**	**455.75**	**1079.97 ± 340.71**	**1227.99 ± 244.16**	**32.35 ± 2.64**	**30.99 ± 2.75**	**41.26 ± 2.56**	**39.15 ± 11.23**	**62.51 ± 2.16**	**62.06 ± 1.73**	**66.86 ± 1.29**	62.58 ± 1.43^*^	**64.19 ± 1.44**	**63.03 ± 2.17**
16:2 n-6	–	–	0.10 ± 0.02	0.40 ± 0.15	0.01 ± 0.00	0.01 ± 0.01	0.003 ± 0.000	0.020 ± 0.010^*^	0.01 ± 0.00	0.01 ± 0.01	0.01 ± 0.00	0.01 ± 0.00^*^	0.003 ± 0.000	0.02 ± 0.01^#^
LA	25.83	95.00	329.16 ± 24.65	870.96 ± 105.11^#^	0.38 ± 0.08	0.72 ± 0.08^#^	6.40 ± 0.46	11.56 ± 2.34^*^	3.02 ± 0.28	7.46 ± 0.10^#^	3.42 ± 0.51	7.51 ± 0.31^#^	3.16 ± 0.17	7.51 ± 0.15^#^
20:2 n-6	0.89	0.40	3.39 ± 0.48	8.88 ± 2.05^#^	0.08 ± 0.02	0.15 ± 0.03^*^	0.09 ± 0.01	0.17 ± 0.05^*^	0.07 ± 0.01	0.14 ± 0.03^#^	0.05 ± 0.01	0.11 ± 0.02^#^	0.05 ± 0.01	0.11 ± 0.02^#^
DGLA	0.26	1.25	29.86 ± 5.51	34.00 ± 13.69	0.35 ± 0.04	0.38 ± 0.01	1.35 ± 0.11	0.65 ± 0.17^#^	0.08 ± 0.02	0.06 ± 0.00^*^	0.05 ± 0.01	0.03 ± 0.02^*^	0.07 ± 0.01	0.04 ± 0.01^*^
ARA	0.48	1.75	140.57 ± 30.17	313.94 ± 49.40^#^	7.93 ± 0.61	8.11 ± 0.64	8.19 ± 0.87	6.28 ± 2.83	0.10 ± 0.03	0.10 ± 0.02	0.06 ± 0.02	0.04 ± 0.03	0.11 ± 0.02	0.07 ± 0.02^*^
AdA	0.11	0.75	1.37 ± 0.20	2.81 ± 0.534^*^	2.06 ± 0.25	2.01 ± 0.25	0.14 ± 0.02	0.17 ± 0.05	0.02 ± 0.01	0.01 ± 0.00^*^	0.01 ± 0.01	0.01 ± 0.00	0.01 ± 0.01	0.01 ± 0.00
**PUFA n-6**	**27.76**	**103.13**	**509.63 ± 50.28**	**1232.70 ± 99.52**^**#**^	**11.22 ± 0.96**	**11.52 ± 0.64**	**16.62 ± 1.38**	**18.93 ± 5.24**	**3.30 ± 0.32**	**7.79 ± 0.09**^**#**^	**3.61 ± 0.53**	**7.72 ± 0.23**^**#**^	**3.42 ± 0.18**	**7.77 ± 0.19**^**#**^
ALA	0.07	0.25	5.24 ± 1.80	9.52 ± 2.24^*^	0.02 ± 0.01	0.02 ± 0.01	0.10 ± 0.03	0.18 ± 0.06^*^	0.01 ± 0.01	0.01 ± 0.01	0.01 ± 0.00	0.01 ± 0.01	0.01 ± 0.01	0.01 ± 0.00
ETA	–	–	2.53 ± 0.43	2.37 ± 0.65	–	–	0.06 ± 0.01	0.03 ± 0.01^*^	0.01 ± 0.00	0.01 ± 0.00	0.01 ± 0.00	0.01 ± 0.01	0.01 ± 0.00	0.01 ± 0.00
EPA	0.19	0.50	39.66 ± 9.47	18.94 ± 2.29^#^	0.33 ± 0.03	0.15 ± 0.02 ^#^	1.71 ± 0.12	0.31 ± 0.11^#^	0.12 ± 0.02	0.03 ± 0.00^#^	0.13 ± 0.02	0.05 ± 0.04^#^	0.12 ± 0.02	0.02 ± 0.01^#^
DPA	0.19	0.63	2.61 ± 0.39	7.59 ± 1.90^#^	0.36 ± 0.05	0.31 ± 0.06	0.23 ± 0.04	0.32 ± 0.09	0.03 ± 0.01	0.02 ± 0.01	0.02 ± 0.01	0.01 ± 0.00	0.03 ± 0.01	0.01 ± 0.00^#^
DHA	0.04	0.25	28.40 ± 6.74	68.66 ± 7.68^#^	8.91 ± 0.57	9.71 ± 0.78	2.36 ± 0.29	2.85 ± 1.28	0.03 ± 0.01	0.01 ± 0.01^*^	0.02 ± 0.00	0.01 ± 0.01	0.02 ± 0.00	0.01 ± 0.00
**PUFA n-3**	**0.48**	**1.63**	**78.43 ± 17.61**	108.08 ± 8.18^*^	**9.62 ± 0.60**	**10.19 ± 0.79**	**4.46 ± 0.37**	**3.69 ± 1.50**	**0.20 ± 0.04**	**0.08 ± 0.01**^**#**^	**0.19 ± 0.01**	0.10 ± 0.06^*^	**0.18 ± 0.02**	**0.06 ± 0.02**^**#**^

*p*-value from t-test: * < 0.05, # < 0.001, AdA – adrenic acid (22:4 n-6), ALA – α-linolenic acid (18:3 n-3), ARA – arachidonic acid (20:4 n-6), DGLA –dihomo-γ-linolenic acid (20:3 n-6), DHA – docosahexaenoic acid (22:6 n-3), DPA – docosapentaenoic acid (22:5 n-3), EPA – eicosapentaenoic acid (20:5 n-3), ETA – eicosatetraenoic acid (20:4 n-3), LA – linoleic acid (18:2 n-6), MUFA – monounsaturated fatty acids, PUFA – polyunsaturated fatty acids, SFA – saturated fatty acid

Boldface, major groups of fatty acid

2. Correct calculations in Table 2 Composition of polyunsaturated FAs in major tissues;

**Table 2:** Composition of polyunsaturated FAs in major tissues

	Chow [mg/g]	Serum [mg/L]	Brain [%]	Liver [%]	Retroperitoneal adipose tissue [%]	Epididymal adipose tissue [%]	Subcutaneous adipose tissue [%]							
	10%	60%	SD	HFD	SD	HFD	SD	HFD	SD	HFD	SD	HFD	SD	HFD
**SFA**	**17.9 ± 0.64**	136 ± 8.00^**^	**1049 ± 286**	1855 ± 397^**^	**46.13 ± 1.06**	**46.82 ± 2.14**	**37.41 ± 0.86**	**38.03 ± 4.72**	**33.75 ± 2.46**	29.73 ± 1.64^*^	**29.08 ± 1.53**	**29.28 ± 1.63**	**31.99 ±1.39**	28.80 ±.29^*^
**MUFA**	**10.7 ± 0.12**	**87.9 ± 4.15**^**#**^	**1164 ± 389**	**1289 ± 274**	**32.35 ± 2.64**	**30.99 ± 2.75**	**41.26 ± 2.56**	**39.15 ± 11.23**	**62.51 ± 2.16**	**62.06 ± 1.73**	**66.86 ± 1.29**	62.58 ± 1.43^*^	**64.19 ± 1.44**	**63.03 ±2.17**
16:2 n-6	0.067 ± 0.003	0.51 ± 0.02^#^	0.04 ± 0.06	0.40 ± 0.17^**^	0.01 ± 0.00	0.01 ± 0.01	0.003 ± 0.000	0.020 ± 0.010^*^	0.01 ± 0.00	0.01 ± 0.01	0.01 ± 0.00	0.01 ± 0.00^*^	0.003 ± 0.000	0.02 ±0.01^#^
LA	2.04 ± 0.04	18.6 ± 0.70^#^	356 ± 29.1	915 ± 114^#^	0.38 ± 0.08	0.72 ± 0.08^#^	6.40 ± 0.46	11.56 ± 2.34^*^	3.02 ± 0.28	7.46 ± 0.10^#^	3.42 ± 0.51	7.51 ± 0.31^#^	3.16 ± 0.17	7.51 ± 0.15^#^
20:2 n-6	0.058 ± 0.009	0.57 ± 0.07^**^	3.78 ± 0.63	9.22 ± 2.37^**^	0.08 ± 0.02	0.15 ± 0.03^*^	0.09 ± 0.01	0.17 ± 0.05^*^	0.07 ± 0.01	0.14 ± 0.03^#^	0.05 ± 0.01	0.11 ± 0.02^#^	0.05 ± 0.01	0.11 ± 0.02^#^
DGLA	0.012 ± 0.0002	0.11 ± 0.01^**^	32.4 ± 6.83	36.0 ± 16.2	0.35 ± 0.04	0.38 ± 0.01	1.35 ± 0.11	0.65 ± 0.17^#^	0.08 ± 0.02	0.06 ± 0.00^*^	0.05 ± 0.01	0.03 ± 0.02^*^	0.07 ± 0.01	0.04 ± 0.01^*^
ARA	0.037 ± 0.004	0.36 ± 0.01^#^	152 ± 37.4	330 ± 59.2^#^	7.93 ± 0.61	8.11 ± 0.64	8.19 ± 0.87	6.28 ± 2.83	0.10 ± 0.03	0.10 ± 0.02	0.06 ± 0.02	0.04 ± 0.03	0.11 ± 0.02	0.07 ± 0.02^*^
AdA	0.008 ± 0.002	0.11 ± 0.02^*^	1.48 ± 0.35	2.81 ± 0.60^#^	2.06 ± 0.25	2.01 ± 0.25	0.14 ± 0.02	0.17 ± 0.05	0.02 ± 0.01	0.01 ± 0.00^*^	0.01 ± 0.01	0.01 ± 0.00	0.01 ± 0.01	0.01 ± 0.00
**PUFA n-6**	**2.16 ± 0.03**	**19.7 ± 0.64**^**#**^	**552 ± 63.9**	**1295 ± 105**^**#**^	**11.22 ± 0.96**	**11.52 ± 0.64**	**16.62 ± 1.38**	**18.93 ± 5.24**	**3.30 ± 0.32**	**7.79 ± 0.09**^**#**^	**3.61 ± 0.53**	**7.72 ± 0.23**^**#**^	**3.42 ± 0.18**	**7.77 ± 0.19**^**#**^
ALA	0.005 ± 0.002	0.049 ± 0.002^#^	5.74 ± 2.18	9.97 ± 2.55^*^	0.02 ± 0.01	0.02 ± 0.01	0.10 ± 0.03	0.18 ± 0.06^*^	0.01 ± 0.01	0.01 ± 0.01	0.01 ± 0.00	0.01 ± 0.01	0.01 ± 0.01	0.01 ± 0.00
ETA	0.002 ± 0.001	0.01 ± 0.01	2.87 ± 0.56	3.44 ± 0.89	–	–	0.06 ± 0.01	0.03 ± 0.01^*^	0.01 ± 0.00	0.01 ± 0.00	0.01 ± 0.00	0.01 ± 0.01	0.01 ± 0.00	0.01 ± 0.00
EPA	0.008 ± 0.002	0.074 ± 0.004^#^	42.9 ± 11.7	20.1 ± 2.77^**^	0.33 ± 0.03	0.15 ± 0.02 ^#^	1.71 ± 0.12	0.31 ± 0.11^#^	0.12 ± 0.02	0.03 ± 0.00^#^	0.13 ± 0.02	0.05 ± 0.04^#^	0.12 ± 0.02	0.02 ± 0.01^#^
DPA	0.016 ± 0.003	0.15 ± 0.007^#^	2.81 ± 0.53	7.87 ± 2.28^**^	0.36 ± 0.05	0.31 ± 0.06	0.23 ± 0.04	0.32 ± 0.09	0.03 ± 0.01	0.02 ± 0.01	0.02 ± 0.01	0.01 ± 0.00	0.03 ± 0.01	0.01 ± 0.00^#^
DHA	0.006 ± 0.003	0.06 ± 0.02	30.9 ± 8.24	72.1 ± 8.81	8.91 ± 0.57	9.71 ± 0.78	2.36 ± 0.29	2.85 ± 1.28	0.03 ± 0.01	0.01 ± 0.01^*^	0.02 ± 0.00	0.01 ± 0.01	0.02 ± 0.00	0.01 ± 0.00
**PUFA n-3**	**0.04 ± 0.01**	**0.35 ± 0.007**^**#**^	**85.2 ± 21.7**	114 ± 9.01^*^	**9.62 ± 0.60**	**10.19 ± 0.79**	**4.46 ± 0.37**	**3.69 ± 1.50**	**0.20 ± 0.04**	**0.08 ± 0.01**^**#**^	**0.19 ± 0.01**	0.10 ± 0.06^*^	**0.18 ± 0.02**	**0.06 ± 0.02**^**#**^

*p*-value from paired t-test: * < 0.05, # < 0.001, AdA – adrenic acid (22:4 n-6), ALA – α-linolenic acid (18:3 n-3), ARA – arachidonic acid (20:4 n-6), DGLA –dihomo-γ-linolenic acid (20:3 n-6), DHA – docosahexaenoic acid (22:6 n-3), DPA – docosapentaenoic acid (22:5 n-3), EPA – eicosapentaenoic acid (20:5 n-3), ETA – eicosatetraenoic acid (20:4 n-3), LA – linoleic acid (18:2 n-6), MUFA – monounsaturated fatty acids, PUFA – polyunsaturated fatty acids, SFA – saturated fatty acid

Boldface, major groups of fatty acid
